# Emerging Trends in Pet Food: Scientific Innovations, Patent Landscapes, and Global Market Development

**DOI:** 10.3390/ani16111753

**Published:** 2026-06-05

**Authors:** Sujira Vuthisopon, Pitiya Kamonpatana, Khwanchat Promhuad, Atcharawan Srisa, Phanwipa Wongphan, Anusorn Seubsai, Phatthranit Klinmalai, Nathdanai Harnkarnsujarit

**Affiliations:** 1 College of Innovation and Industrial Management, King Mongkut’s Institute of Technology Ladkrabang (KMITL), Bangkok 10520, Thailand; sujira.vu@kmitl.ac.th; 2 Department of Food Science and Technology, Faculty of Agro-Industry, Kasetsart University, Bangkok 10900, Thailand; fagipyk@ku.ac.th; 3 Department of Packaging and Materials Technology, Faculty of Agro-Industry, Kasetsart University, Bangkok 10900, Thailand; khwanchatpromhuad@gmail.com (K.P.); atcharawan.sri@ku.th (A.S.); phanwipa.w@ku.th (P.W.); 4 Department of Chemical Engineering, Faculty of Engineering, Kasetsart University, Bangkok 10900, Thailand; fengasn@ku.ac.th; 5 Faculty of Agro-Industry, Chiang Mai University, Samut Sakhon 74000, Thailand; 6 Center for Advanced Studies for Agriculture and Food (CASAF), Kasetsart University Institute for Advanced Studies (KUIAS), Kasetsart University, 50 Ngam Wong Wan Rd., Latyao, Chatuchak, Bangkok 10900, Thailand

**Keywords:** functional, pet food, companion animals, canine, feline

## Abstract

The pet food sector has progressively evolved over the past decade from conventional nutrition toward functionally targeted and sustainability-oriented systems that are increasingly parallel developments in human health. While numerous reviews have examined individual aspects of pet food innovation, an integrated perspective linking scientific research, patent activity, and global market dynamics remains limited. This review addresses this gap by systematically synthesizing peer-reviewed literature, patent landscapes, and product launch data to identify key drivers and bottlenecks shaping contemporary pet food innovation. The analysis highlights a strong concentration of research and patent activity in health-oriented functional formulations, particularly those targeting gastrointestinal health, immune modulation, and age-related conditions, while postbiotics, precision nutrition, and digital tools remain comparatively underdeveloped. Sustainability-driven ingredients and alternative proteins show growing momentum but face persistent challenges related to scalability, regulation, and sensory acceptance. The commercial success of functional pet foods depends on translating scientific findings into stable, manufacturable, and evidence-supported products. Future innovation will therefore be shaped by technologies that connect biological function with process feasibility and market readiness. This review concludes that future progress in pet food innovation will depend on integrated frameworks that align biological efficacy, technological feasibility, and market viability, thereby bridging the gap between scientific advancement and commercial implementation.

## Simple Summary

The pet food industry is changing from basic nutrition toward functional nutrition focused on long-term health benefits. However, scientific research, patents, and commercial products are often developed in isolation, limiting a comprehensive perspective that connects knowledge to practical application. This review integrates evidence from scientific publications, intellectual property databases, and global product launch trends to reflect the development direction, market saturation points, and innovation gaps in pet food. Data analysis shows sustained growth in formulations targeting gut health, immunity, obesity, aging, and alternative proteins. In contrast, machine learning–driven formulation optimization, predictive health modeling, and logistics systems remain at earlier stages of development. This review proposes an integrated framework linking research, technology, and market trends to guide future research priorities, policy development, and the creation of efficient and safe functional pet food products.

## 1. Introduction

**Figure 1:**
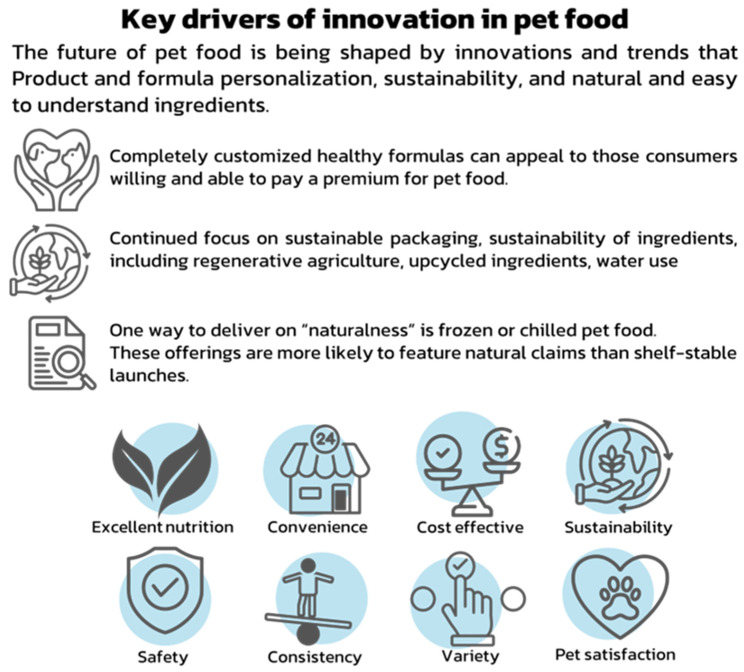
Key drivers of innovation in pet food. The figure summarizes the major drivers influencing pet food research and product development, including sustainability, natural and functional ingredients, product safety and quality, market expansion, consumer preferences, animal health and well-being, and the growing humanization of companion animals.

The global pet food industry has evolved well beyond its traditional role of providing basic nutrition, emerging as a highly dynamic sector shaped by advances in nutritional science, shifting consumer behavior, and rapid technological innovation. This transformation is largely driven by the growing humanization of pets, whereby companion animals are increasingly regarded as family members with complex health, wellness, and longevity needs. Consequently, pet owners now demand products that mirror trends in human nutrition, including functional and preventive health benefits, clean-label formulations, sustainability, and personalized dietary solutions (Figure 1).

This paradigm shift has stimulated sustained research interest in health-oriented pet food formulations designed not only to meet nutritional requirements but also to support physiological functions, delay the onset or progression of nutrition-related disorders, and improve overall quality of life. Functional pet foods defined as products incorporating bioactive compounds, nutraceuticals, or condition specific ingredients have evolved as a major and increasingly diversified area of innovation, building on long-standing clinical nutrition frameworks. Current scientific literature consistently highlights digestive health, immune modulation, antioxidant capacity, and joint support as dominant research themes, closely paralleling developments in human functional nutrition. In particular, the gut microbiome has become a focal point of pet nutrition research, with accumulating evidence linking intestinal health to systemic outcomes such as immune competence, metabolic regulation, and even behavior [1,2,3]. In parallel, the rapid expansion of digital technologies including artificial intelligence (AI), machine learning, and predictive modeling has begun to reshape pet food research and product development. These tools offer new opportunities to optimize formulations, personalize feeding strategies, and model biological responses with greater precision, thereby accelerating innovation and improving consumer alignment [4,5]. Nevertheless, despite their growing visibility, the application of digital technologies in the pet food sector remains largely confined to research and development activities, such as formulation optimization and predictive nutrient modeling. Consequently, commercial scale has yet to be systematically assessed.

Sustainability has long been a defining driver of innovation within the pet food sector, with its influence expanding in scope and strategic importance in recent years. Heightened awareness of environmental impacts associated with conventional animal-based proteins has prompted exploration of alternative protein sources, such as insects, algae, and plant-based ingredients, as well as upcycled raw materials aligned with circular economy principles [6,7]. While these approaches show considerable promise, their widespread adoption is constrained by regulatory uncertainties, sensory acceptance, nutritional validation, and scalability challenges, underscoring the need for coordinated scientific and industrial strategies. As formulations grow increasingly complex, innovation in sensory optimization and packaging technologies has likewise gained prominence. Palatability remains a critical determinant of product success, particularly when functional or novel ingredients introduce undesirable flavors, textures, or aromas [8,9]. Concurrently, advances in packaging play a pivotal role in preserving product quality, extending shelf life, and supporting emerging distribution models such as direct-to-consumer and e-commerce channels in a globalized marketplace.

Despite the rapid expansion of research, patent filings, and commercial activity in the pet food sector, much of the existing literature remains thematically segmented. Previous reviews have provided valuable insights into specific areas, such as functional ingredients and nutraceuticals, microbiome-related nutrition, alternative protein sources, palatability technologies, or consumer and market trends. However, these studies typically examine scientific, technological, or market dimensions in isolation, without systematically linking research outputs to patent activity and global commercialization patterns. As a result, a comprehensive understanding of how scientific advances translate into protected technologies and market implementation remains limited.

In this context, the present review advances the field by offering an integrated synthesis of peer-reviewed research, patent landscapes, and global product launch data related to functional pet food innovation. By jointly analyzing health-focused formulations, sustainability-driven ingredients, digitalization, sensory optimization, and personalization within a single framework, this review identifies cross-domain innovation drivers, technological bottlenecks, and translational gaps that are not evident from single-perspective analyses. This integrative approach provides a clearer roadmap for researchers, industry stakeholders, and policymakers navigating the evolving pet food landscape.

## 2. Methodology

This review adopted an integrative landscape-analysis approach to examine trends in scientific research, patent activity, and commercial product development related to pet food innovation. Three complementary data sources were used: Scopus for scientific publications, the World Intellectual Property Organization (WIPO) PA-TENTSCOPE database for patent activity, and Mintel’s Global New Products Database (GNPD) for commercial product launches.

Data collection was conducted using combinations of general pet food terms, including “pet food”, “dog food”, “cat food”, “canine”, “feline”, “companion animal nutrition”, and “companion animal food”, together with theme-specific keywords related to functional health benefits, probiotics and microbiome, precision nutrition, artificial intelligence, sustainability, sensory technologies, alternative proteins, logistics, and supply-chain management. The specific keyword combinations used for each thematic analysis are reported in the corresponding figure captions. Because several thematic domains are conceptually related, overlap between categories was expected and accepted within the landscape-analysis framework. Individual publications, patents, or product records could therefore contribute to more than one thematic category when multiple relevant keywords were present. Consequently, the results were interpreted as indicators of relative thematic activity rather than mutually exclusive classifications. The thematic categories were developed to reflect major innovation domains repeatedly identified across the scientific literature, patent landscape, and commercial product databases. The classification framework was intended to facilitate cross-dataset trend interpretation among research activity, technological development, and market implementation. Alternative classification approaches may produce different category structures; however, the selected framework captured the most consistently recurring innovation themes across all three datasets. The analysis covered the period from January 2016 to December 2025. For scientific publications, searches were performed in Scopus using English-language records. Peer-reviewed journal articles, reviews, conference papers, and relevant indexed documents were considered. The objective was to evaluate broad innovation patterns rather than generate a systematic evidence synthesis, the analysis focused on relative publication trends rather than the final number of unique documents retained for each topic.

Patent data were collected from the World Intellectual Property Organization (WIPO) PATENTSCOPE database. Searches combined general pet food terms with theme-specific keywords and were applied to English-language patent titles, abstracts, and claims. Patent publication dates were limited to January 2016–December 2025. Because the analysis relied primarily on keyword-based retrieval, some relevant patents may not have been captured, and certain patents may have been represented across multiple technology themes. Therefore, patent counts were interpreted as comparative indicators of technological activity rather than exhaustive patent inventories.

Commercial product launch trends were assessed using Mintel GNPD. Product records were filtered using the category “Pet Food” together with relevant dog and cat food subcategories. Product launches between January 2016 and December 2025 were included. Mintel GNPD data were used to evaluate market implementation of technologies identified in the publication and patent analyses. This approach enabled comparison between scientific activity, technological development, and commercial adoption across major innovation themes. Publication counts, patent records, and product launch data were not directly compared as equivalent quantitative measures. Each dataset was used to represent a different stage of the innovation pathway, with publications reflecting scientific activity, patents representing technological development, and product launches indicating commercial implementation. Trend comparisons were therefore interpreted qualitatively to identify areas of convergence, divergence, and translational gaps across the pet food innovation landscape.

The purpose of this study was to identify broad innovation patterns, translational gaps, and emerging opportunities within the pet food sector. Accordingly, the review was designed as a narrative landscape analysis rather than a systematic review or formal bibliometric study. Detailed search terms used for each thematic analysis are provided in the corresponding figure captions.

## 3. Trend Analysis of Pet Food Research and Innovation

**Figure 2:**
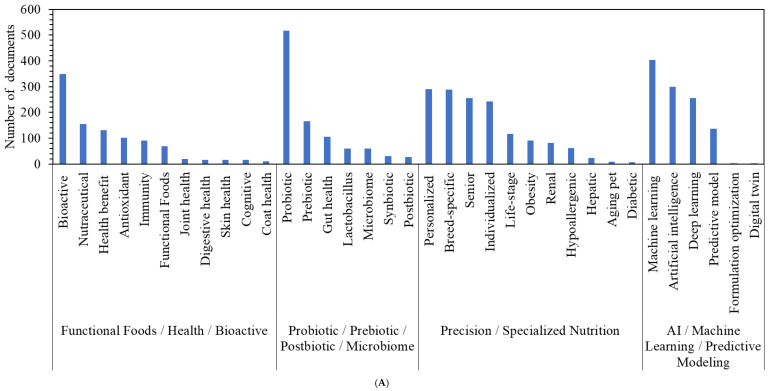

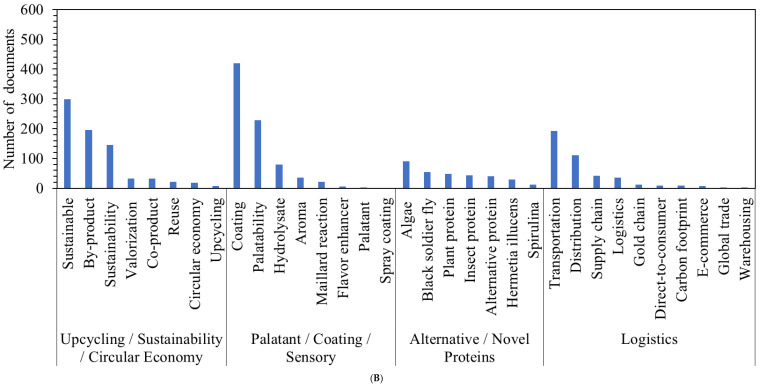
Number of publications indexed in the Scopus database on functional pet food research from 2000 to 2025, categorized into major research themes. Panel (**A**) shows research related to functional foods, health, and bioactive ingredients; probiotics, prebiotics, postbiotics, and microbiome-related topics; precision and specialized nutrition; and artificial intelligence and predictive modeling. Panel (**B**) shows research related to sustainability and upcycling; palatability, coating, and sensory technologies; alternative and novel protein sources; and logistics and supply chain. The data were obtained using Scopus searches with combinations of general pet food terms (e.g., “pet food”, “dog food”, “cat food”, “companion animal nutrition”) together with theme-specific keywords related to health functions, microbiome, precision nutrition, artificial intelligence, sustainability, sensory technologies, alternative proteins, and logistics.

Figure 2 provides a comparative overview of publication and patent activity across major thematic domains in functional pet food research, allowing assessment of relative research maturity rather than absolute innovation novelty. The strong concentration of documents related to functional foods and health-oriented bioactive reflects a historically well-established research axis, where scientific knowledge, regulatory familiarity, and ingredient availability have supported sustained investigation and commercialization. In contrast, lower document densities in emerging domains highlight uneven research development and suggest areas where scientific validation, technological feasibility, or translational pathways remain limited. Thus, Figure 2 serves as a baseline framework for identifying both saturation points in established research areas and underexplored domains that warrant more critical evaluation in subsequent analyses.

A substantial body of work is also observed in the probiotic, prebiotic, postbiotic, and microbiome category. High document counts for probiotics and prebiotics indicate that modulation of gut microbiota remains a central strategy for improving pet health, while comparatively lower numbers for postbiotics and microbiome-focused approaches suggest that these areas are still emerging. This imbalance implies a transition phase in which mechanistic understanding of host–microbiome interactions is beginning to translate into applied pet food innovations [2,3].

The precision and specialized nutrition cluster shows strong representation, particularly for personalized and breed-specific nutrition concepts. This trend highlights a shift from generalized formulations toward targeted nutritional solutions tailored to life stage, genetic background, and health status. In contrast, niche topics such as hypoallergenic or hepatic diets display lower document frequencies, suggesting specialized but narrower application scopes [10,11].

In the AI, machine learning, and predictive modeling category, document counts remain moderate but non-negligible. Keywords such as machine learning, artificial intelligence, and formulation optimization demonstrate growing interest in data-driven approaches for pet food design, although their prevalence is still markedly lower than traditional ingredient-based strategies. This indicates that digital tools are currently positioned as enabling technologies rather than primary innovation drivers [4,12].

The upcycling, sustainability, and circular economy category exhibits a distinct pattern, with upcycling and sustainability-related keywords showing relatively high activity compared with co-product reuse or circular economy terminology. This suggests that sustainability in pet food research is primarily framed around ingredient sourcing and waste valorization rather than fully integrated circular systems [6,13].

Research related to palatants, coatings, and sensory enhancement occupies a mid-range position. High counts for palatability and coating indicate that sensory acceptance remains a critical factor in functional pet food success, particularly when health-promoting ingredients may negatively affect taste or texture. More chemistry-driven approaches, such as Maillard reaction-based flavor generation, appear less frequently, implying higher technical barriers or more specialized use [8].

Finally, the alternative and novel protein category, including insect protein, plant protein, and microbial protein, shows growing but uneven activity. Insect protein demonstrates notable representation, reflecting its dual role in sustainability and functional nutrition, whereas microbial and fermentation-derived proteins remain less explored [6]. The logistics-related keywords display the lowest document counts overall, indicating that innovation efforts are currently concentrated upstream at the ingredient and formulation levels rather than downstream distribution and supply chain optimization [14].

### 3.1. Temporal Trends in Functional Pet Food Research (2016–2025)

**Figure 3:**
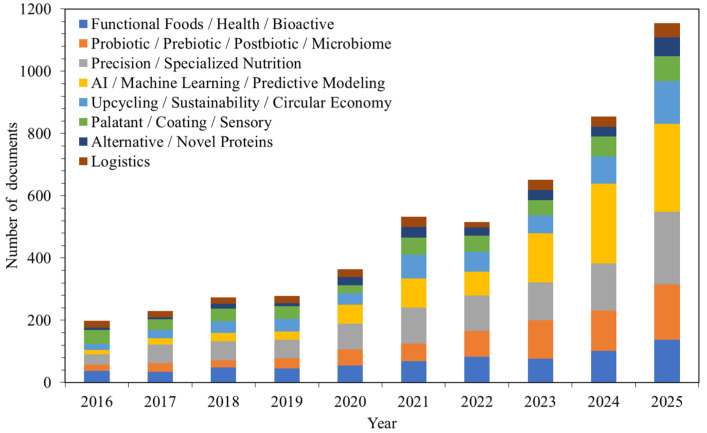
Annual number of publications indexed in the Scopus database on functional pet food research from 2016 to 2025, classified by major research themes: functional foods, health, and bioactive ingredients; probiotic, prebiotic, postbiotic, and microbiome research; precision and specialized nutrition; artificial intelligence and predictive modeling; sustainability and upcycling; palatability, coating, and sensory technologies; alternative and novel protein sources; and logistics and supply chain. The data were obtained from Scopus using general pet food terms, including “pet food”, “dog food”, “cat food”, “canine”, “feline”, “companion animal nutrition”, and “companion animal food”, combined with theme-specific keywords related to health benefits, microbiome modulation, precision nutrition, artificial intelligence, sustainability, sensory technologies, alternative proteins, and logistics.

Figure 3 presents the annual evolution of publications indexed in Scopus related to functional pet food from 2016 to 2025, categorized by major thematic domains. A pronounced upward trend is observed across all categories, with total publication numbers increasing steadily and accelerating markedly after 2020. This pattern reflects heightened academic and industrial interest in companion animal nutrition, driven by pet humanization, aging pet populations, and growing demand for health-oriented pet food solutions [13,15,16].

Throughout the entire period, functional foods, health, and bioactive-related studies consistently represent the largest share of publications. This dominance underscores the central role of health benefit–driven research in pet food science, particularly in areas such as immunity, digestive health, skin and coat condition, joint health, and antioxidant activity. The sustained growth of this category suggests that functional ingredient development remains the foundational pillar of innovation in the field, consistent with earlier observations in both companion animal and human nutrition research [2,3].

The probiotic, prebiotic, postbiotic, and microbiome category exhibited a continuous increase, with publication counts approximately doubling between 2016 and 2025. This trend indicates a shift toward gut health–centered nutritional strategies and reflects expanding recognition of the gut–immune and gut–metabolic axes in companion animals. While probiotics and prebiotics dominate early publications, the gradual rise of postbiotic- and microbiome-related keywords suggests increasing mechanistic depth and maturation of the research landscape [1,2,3].

Publications related to precision and specialized nutrition show one of the most rapid growth rates over the examined period. Concepts such as personalized nutrition, breed-specific diets, life-stage targeting, and management of chronic conditions (e.g., obesity, renal or hepatic disorders) gain momentum after 2020. This acceleration highlights a transition from generalized formulations toward tailored nutritional interventions, aligning pet nutrition more closely with precision nutrition paradigms established in human health research [10,11,17].

In contrast, AI, machine learning, and predictive modeling remained lower in publication volume than ingredient-focused categories but showed a noticeable increase after 2021. Although absolute publication numbers remain lower than ingredient-focused categories, the steep growth trajectory suggests increasing adoption of data-driven tools for formulation optimization, predictive health modeling, and digital decision-support systems. These technologies appear to function primarily as enablers that enhance formulation efficiency and personalization rather than as standalone innovation drivers [4,12].

In contrast, AI, machine learning, and predictive modeling emerge later but demonstrate a sharp rise from 2021 onward. Although absolute publication numbers remain lower than ingredient-focused categories, the steep growth trajectory suggests increasing adoption of data-driven tools for formulation optimization, predictive health modeling, and digital decision-support systems. These technologies appear to function primarily as enablers that enhance formulation efficiency and personalization rather than as standalone innovation drivers [4,12].

The upcycling, sustainability, and circular economy category shows steady growth, particularly after 2020, reflecting broader societal and regulatory pressures toward sustainable food systems. Increased attention to by-product utilization, valorization, and sustainable sourcing indicates that environmental considerations are becoming progressively integrated into functional pet food research, although they remain secondary to health-focused themes [6,13].

Research on palatants, coatings, and sensory enhancement maintains moderate but consistent growth. This reflects the persistent need to balance functional efficacy with palatability, especially as formulations incorporate bioactive or alternative ingredients that may negatively affect sensory acceptance. The continued presence of this category emphasizes that sensory performance remains a critical determinant of commercial success in functional pet food development [8,16].

Finally, alternative and novel protein sources, including insect proteins, algae, and plant-based proteins, display gradual yet accelerating growth toward the later years of the period. This trend highlights increasing exploration of sustainable protein alternatives driven by environmental and resource-efficiency concerns, although publication volumes suggest that these approaches are still emerging compared with conventional animal-derived ingredients [6,13]. In contrast, the logistics and supply chain category remains the smallest contributor throughout the timeline, indicating that innovation efforts remain primarily concentrated on upstream formulation and ingredient development rather than downstream distribution or supply chain optimization [14].

#### 3.1.1. Evolution of Health-Oriented in Functional Pet Food (2016–2025)

**Figure 4:**
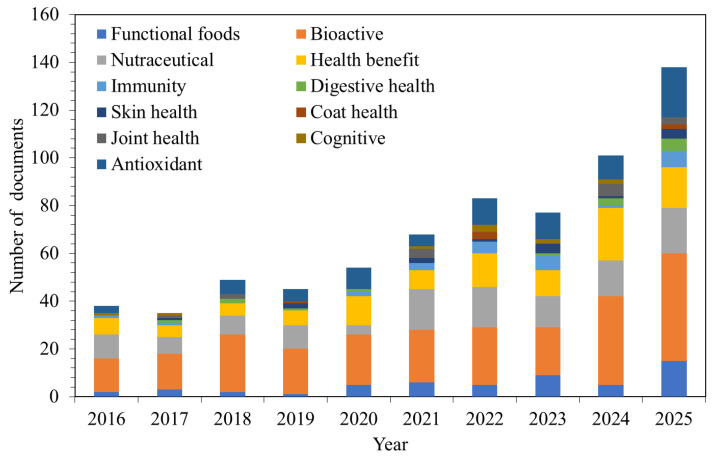
Annual number of publications indexed in the Scopus database on health-oriented functional pet food research from 2016 to 2025, classified by major health-related targets, including general health benefits, immune support, gastrointestinal health, skin and coat health, joint health, cognitive health, and antioxidant-related functions. The data were obtained from Scopus using general pet food terms, including “pet food”, “dog food”, “cat food”, “canine”, “feline”, “companion animal nutrition”, and “companion animal food”, combined with health-related keywords such as “health benefit”, “immunity”, “digestive health”, “skin health”, “coat health”, “joint health”, “cognitive”, and “antioxidant”.

Figure 4 summarizes annual publication trends related to health-oriented functional pet food research from 2016 to 2025, focusing on specific health benefit categories. Research in functional pet food has demonstrated a consistent upward trend, reflecting a growing scientific and commercial emphasis on formulations that promote companion animal health through targeted nutritional strategies [13]. Nutraceuticals and general health claims continue to dominate the landscape, serving as foundational themes for emerging functional categories. Notably, studies on immunity and gastrointestinal health have expanded significantly, underscoring the increasing recognition of the gut–immune axis as a key target for dietary intervention [1]. The steady rise in antioxidant-focused research reflects continued interest in oxidative stress as a common underlying factor in chronic and age-related conditions. Meanwhile, joint health has garnered growing attention, particularly in aging pet populations, aligning with broader demographic shifts and mobility-related concerns. Although still limited in number, cognitive health studies are gradually emerging, likely influenced by human aging research and rising demand for senior pet nutrition [18]. These trends collectively indicate a transition in functional pet food research from general wellness claims toward condition-specific interventions grounded in physiological mechanisms.

#### 3.1.2. Gut Health-Oriented Functional Pet Food (2016–2025)

**Figure 5:**
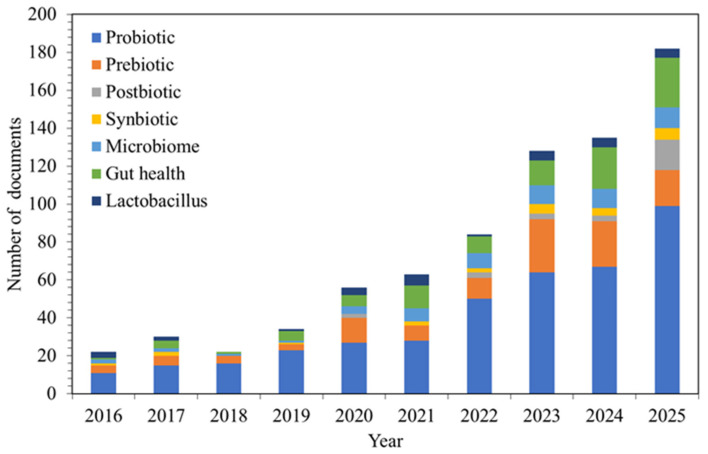
Annual number of publications indexed in the Scopus database on gut health–oriented functional pet food research from 2016 to 2025, classified by microbiome-related research approaches, including probiotic, prebiotic, postbiotic, synbiotic, microbiome-focused studies, gut health, and Lactobacillus-related research. The data were obtained from Scopus using general pet food terms, including “pet food”, “dog food”, “cat food”, “canine”, “feline”, “companion animal nutrition”, and “companion animal food”, combined with microbiome-related keywords such as “probiotic”, “prebiotic”, “postbiotic”, “synbiotic”, “microbiome”, “gut health”, and “Lactobacillus” to reflect research trends in gut microbiome–related functional nutrition.

The rising interest in gut health-focused functional pet food research (Figure 5) is evidenced by a continuous increase in publications from 2016 to 2025, particularly accelerating after 2020, driven by a growing understanding of the gut’s role in systemic health and disease prevention in companion animals [1]. Probiotics dominate the literature, reflecting their widespread investigation and commercial relevance rather than uniformly established efficacy. While numerous studies report associations between probiotic supplementation and modulation of gastrointestinal microbiota, evidence for consistent improvements in host outcomes, such as nutrient absorption, metabolic balance, or immune function [19,20]. Parallel growth in prebiotic research supports their function as fermentable substrates that enhance probiotic efficacy and endogenous microbiota composition. Synbiotic formulations, although increasingly discussed, remain controversial, as robust evidence for specific, consistently effective probiotic–prebiotic combinations are still limited. Meanwhile, emerging studies on postbiotics and microbiome-level interactions point to a shift toward mechanistic and systems-level understanding of gut ecology, though their application in commercial pet food remains nascent [21]. The steady rise in research using the term “gut health” signals the integration of microbiota science with broader physiological functions, including immune modulation and metabolic regulation. Notably, research specifically targeting Lactobacillus strains remains limited but shows a gradual increase, indicating a trend toward taxonomic and functional specificity aligned with regulatory demands and strain-level efficacy evidence [3].

#### 3.1.3. Emergence of Data-Driven and Digital Technologies in Functional Pet Food (2016–2025)

**Figure 6:**
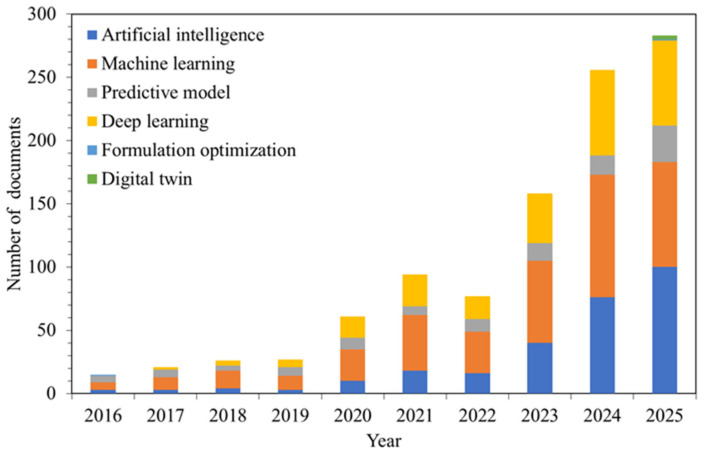
Annual number of publications indexed in the Scopus database on data-driven and digital technologies in functional pet food research from 2016 to 2025, classified by computational and digital approaches, including artificial intelligence, machine learning, predictive modeling, deep learning, formulation optimization, and digital twin technologies. The data were obtained from Scopus using general pet food terms, including “pet food”, “dog food”, “cat food”, “canine”, “feline”, “companion animal nutrition”, and “companion animal food”, combined with digital technology–related keywords such as “artificial intelligence”, “machine learning”, “predictive model”, “deep learning”, “formulation optimization”, and “digital twin”.

The integration of digital technologies into functional pet food research (Figure 6) has expanded notably since 2020, marking a paradigm shift from traditional formulation practices to data-driven and computational approaches. Recent growth in the application of machine learning and deep learning reflects increasing reliance on these tools for optimizing ingredient combinations, predicting nutritional outcomes, and personalizing pet nutrition strategies [22,23]. Artificial intelligence (AI) is emerging as a unifying framework encompassing various analytical techniques, facilitating the interpretation of high-dimensional biological and behavioral data in companion animal research [5]. Predictive modeling, though more established, continues to serve as a foundation for nutrient requirement estimation and diet performance assessment. Meanwhile, digital tools for formulation optimization are gaining traction, enabling precise tailoring of diets to life stage, health status, and functional outcomes. Although still in its infancy within pet nutrition, the digital twin concept—used to simulate biological responses and production environments—represents a promising direction for future research, with potential to enhance efficiency and reproducibility in product development [24]. Although publication trends indicate growing academic interest in artificial intelligence, machine learning, and predictive modeling for pet food applications, most reported uses remain exploratory or supportive in nature. Current studies primarily demonstrate the conceptual potential of data-driven tools for formulation optimization, nutrient prediction, and hypothesis generation, rather than widespread deployment in commercial pet food manufacturing or product personalization. At present, AI-based approaches are more commonly applied as internal decision-support tools within research and development pipelines, while fully integrated, consumer-level AI-driven nutrition systems remain largely absent from mainstream pet food markets.

#### 3.1.4. Sustainability-Oriented in Functional Pet Food (2016–2025)

**Figure 7:**
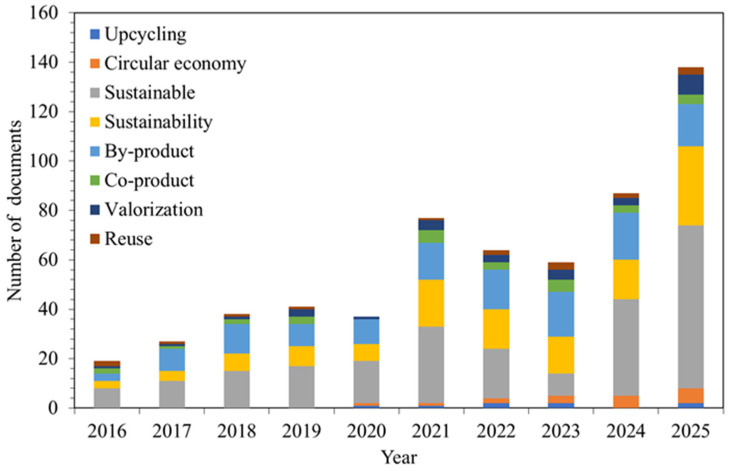
Annual number of publications indexed in the Scopus database on sustainability-oriented research in functional pet food from 2016 to 2025, classified by sustainability-related approaches, including upcycling, circular economy, sustainability concepts, by-product and co-product utilization, valorization, and reuse. The data were obtained from Scopus using general pet food terms, including “pet food”, “dog food”, “cat food”, “canine”, “feline”, “companion animal nutrition”, and “companion animal food”, combined with sustainability-related keywords such as “upcycling”, “circular economy”, “sustainable”, “sustainability”, “by-product”, “co-product”, “valorization”, and “reuse”.

Sustainability has become an increasingly central theme in functional pet food research, as reflected by a steady rise in publications from 2016 to 2025 (Figure 7) and a sharp acceleration after 2020, coinciding with broader environmental awareness and circular economy principles [13]. Among sustainability-driven topics, the consistent prominence of the term “sustainable” in formulation and sourcing practices highlights a shift from peripheral concern to strategic priority within the industry. The growing adoption of sustainability metrics in ingredient selection and processing aligns with consumer demands for environmentally responsible products [7]. Notably, by-product utilization has gained traction as a viable method to reduce food waste and repurpose agricultural residues for nutritional value in pet diets. This trend supports the role of pet food in larger agri-food valorization systems, especially in terms of protein recovery and resource circularity. While concepts such as upcycling and valorization are increasingly discussed, their application in pet food remains nascent, limited by technological and regulatory constraints. In contrast, circular economy and reuse frameworks are underrepresented, indicating that most sustainability efforts remain focused on ingredient-level innovation rather than system-wide transformation [25]. The evolving focus on waste reduction and resource efficiency suggests that functional pet food research is gradually aligning with the global shift toward sustainable, low-impact food systems. In contrast, fully integrated circular economy frameworks such as closed-loop reuse systems, end-of-life material recovery, and coordinated supply-chain circularity remain comparatively underrepresented in the literature. Their limited adoption reflects persistent technological, regulatory, and logistical constraints that extend beyond individual ingredient innovation. Accordingly, current sustainability efforts in functional pet food are best characterized as incremental and ingredient-focused, with system-wide circular models remaining largely aspirational. Nevertheless, the growing emphasis on waste reduction and resource efficiency suggests a gradual alignment with broader goals for sustainable, low-impact food systems.

#### 3.1.5. Palatability Enhancement and Sensory Technologies for Functional Pet Food (2016–2025)

**Figure 8:**
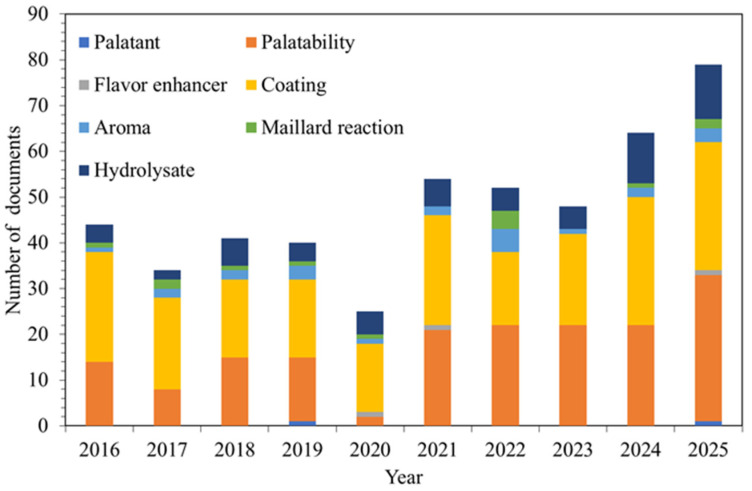
Annual number of publications indexed in the Scopus database on palatability enhancement and sensory technologies in functional pet food research from 2016 to 2025, classified by sensory-related approaches, including palatants, palatability, flavor enhancers, coating technologies, aroma, Maillard reaction, and hydrolysate-based ingredients. The data were obtained from Scopus using general pet food terms, including “pet food”, “dog food”, “cat food”, “canine”, “feline”, “companion animal nutrition”, and “companion animal food”, combined with sensory-related keywords such as “palatant”, “palatability”, “flavor enhancer”, “coating”, “spray coating”, “aroma”, “Maillard reaction”, and “hydrolysate”.

Research on sensory and palatability strategies in functional pet food (Figure 8) has gained increasing momentum between 2016 and 2025, particularly after 2020, reflecting renewed interest in optimizing sensory acceptance as formulations incorporate more bioactive and alternative ingredients. Palatability and coating technologies dominate the literature throughout this period, emphasizing their pivotal role in ensuring voluntary intake and compliance in companion animals, particularly when using less inherently appealing functional ingredients [26]. The post-2021 surge in palatability-focused studies likely reflects the industry’s recognition that efficacy in functional diets must be matched by sensory compatibility. Flavor enhancers and aroma compounds show moderate but steady growth, functioning primarily to mask undesirable notes from novel proteins or supplements. Research on protein hydrolysates also shows gradual increases, highlighting their value as dual-purpose ingredients enhancing palatability while providing digestible peptides and functional amino acids [27]. Although Maillard reaction products are well recognized for their flavor-enhancing effects in commercial settings, academic research in this area remains limited. This restraint may be attributed not only to concerns over nutrient degradation or the formation of undesirable compounds, such as acrylamide, but also to regulatory and safety considerations governing pet food production [28]. In many jurisdictions, including the United States, flavoring ingredients and processing aids must comply with feed safety regulations that require ingredients to be generally recognized as safe or formally approved, and prohibit misleading or drug-like claims on pet food labels. Consequently, sensory innovation in pet food increasingly favors generation approaches that balance palatability enhancement with nutritional integrity, regulatory compliance, and product safety. These trends underscore a growing convergence between functionality, sensory science, and regulatory oversight in pet food innovation.

#### 3.1.6. Emerging Trends in Alternative and Novel Protein Sources for Functional Pet Food (2016–2025)

**Figure 9:**
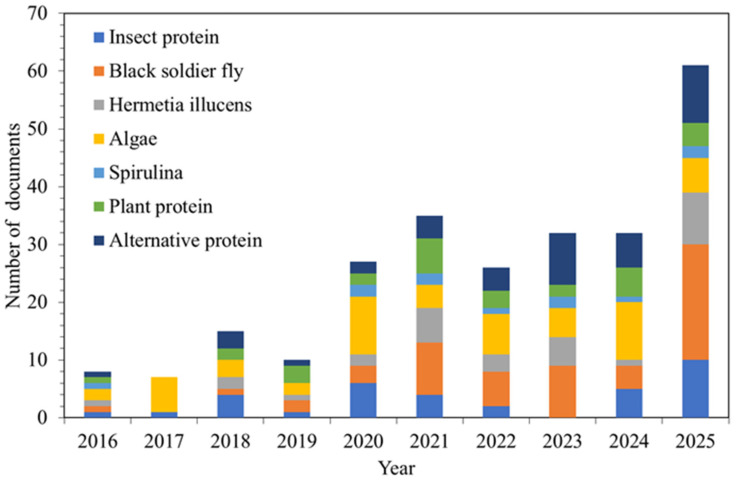
Annual number of publications indexed in the Scopus database on alternative and novel protein sources in functional pet food research from 2016 to 2025, classified by protein-source categories, including insect protein, black soldier fly (*Hermetia illucens*), algae, spirulina, plant protein, and alternative protein. The data were obtained from Scopus using general pet food terms, including “pet food”, “dog food”, “cat food”, “canine”, “feline”, “companion animal nutrition”, and “companion animal food”, combined with alternative protein–related keywords such as “insect protein”, “black soldier fly”, “*Hermetia illucens*”, “algae”, “spirulina”, “plant protein”, and “alternative protein”.

Research into alternative protein sources for functional pet food (Figure 9) has expanded significantly since 2020, reflecting a broader shift toward sustainable, nutritionally viable ingredients for companion animals. Insect-based proteins—especially those derived from the black soldier fly (*Hermetia illucens*)—emerge as the most rapidly growing research focus, owing to their high digestibility, favorable amino acid profile, and low environmental impact [7,29]. The sharp rise in related publications post-2021 suggests increasing acceptance of insect meal as a commercially viable pet food ingredient. Algae and spirulina also demonstrate steady growth, primarily due to their multifunctional properties, including antioxidant activity, immune modulation, and enrichment in essential micronutrients [30,31]. Though less prolific, plant protein–related research has increased gradually, reflecting its role in diversifying protein inputs while managing allergenicity and enhancing sustainability [32]. The rise of integrative studies categorized under “alternative protein” in recent years indicates a shift from single-source evaluations to comparative frameworks assessing the nutritional and environmental performance of diverse protein sources [13]. This trend supports the alignment of functional pet food innovation with broader goals in sustainable food systems and circular resource use.

#### 3.1.7. Logistics and Supply Chain–Related Research in Functional Pet Food (2016–2025)

**Figure 10:**
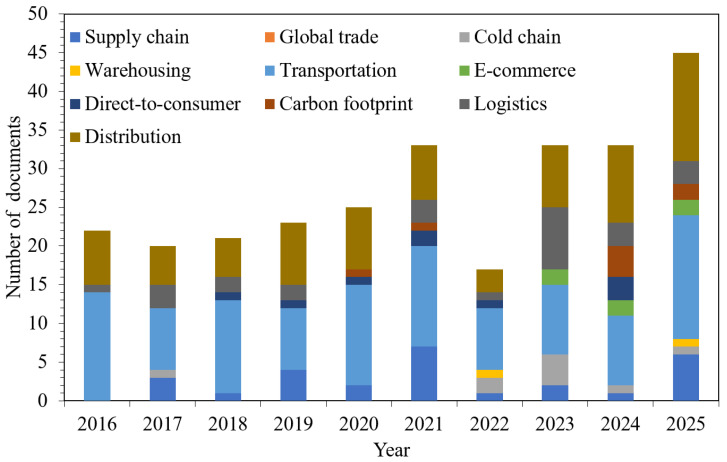
Annual number of publications indexed in the Scopus database on logistics and supply chain research related to functional pet food from 2016 to 2025, classified by distribution and supply chain topics, including supply chain, global trade, cold chain, warehousing, transportation, e-commerce, direct-to-consumer models, carbon footprint, logistics, and distribution. The data were obtained from Scopus using general pet food terms, including “pet food”, “dog food”, “cat food”, “canine”, “feline”, “companion animal nutrition”, and “companion animal food”, combined with logistics-related keywords such as “logistics”, “supply chain”, “distribution”, “global trade”, “cold chain”, “warehousing”, “transportation”, “e-commerce”, “direct-to-consumer”, and “carbon footprint”.

Figure 10 presents annual publication trends related to logistics and supply chain aspects in functional pet food research from 2016 to 2025. This growth reflects a broader recognition of the role that distribution systems play in maintaining product integrity, especially for functional formulations containing sensitive bioactive compounds [13]. Transportation and distribution are the most frequently cited logistics components, highlighting persistent concerns around physical handling, shelf-life stability, and delivery efficiency in globalized supply networks. The rising number of publications referencing “supply chain” and “global trade” after 2020 coincides with heightened industry awareness of supply chain fragility and the need for greater traceability and risk mitigation, especially in the wake of systemic disruptions. Environmental considerations are also increasingly evident, as shown by the emergence of “carbon footprint” as a topic of interest, aligning with broader sustainability goals across the agri-food sector [33]. Additionally, post-2021 trends indicate modest but growing interest in e-commerce and direct-to-consumer models, suggesting a paradigm shift in how functional pet food products reach end-users, spurred by digital transformation and shifting consumer preferences [34]. Collectively, these developments position supply chain research as an essential complement to formulation science, with implications for product performance, accessibility, and environmental responsibility.

#### 3.1.8. Expansion of Precision and Specialized Nutrition in Functional Pet Food Research (2016–2025)

**Figure 11:**
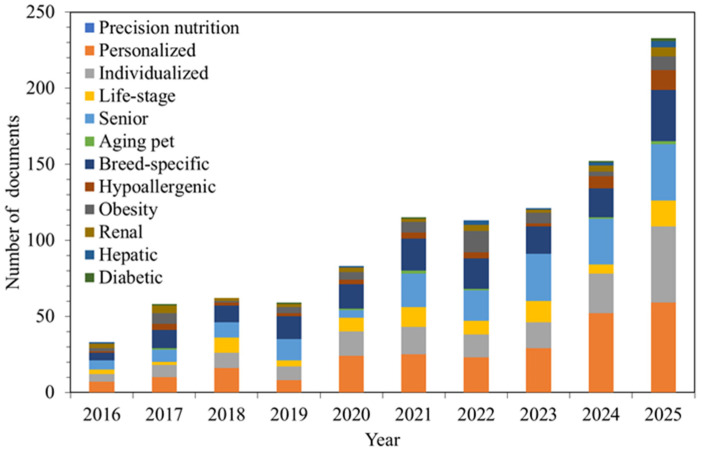
Annual number of publications indexed in the Scopus database on precision and specialized nutrition in functional pet food research from 2016 to 2025, classified by nutrition-targeting approaches, including precision nutrition, personalized and individualized nutrition, life-stage nutrition, senior and aging pet nutrition, breed-specific nutrition, hypoallergenic diets, obesity management, and renal, hepatic, and diabetic nutrition. The data were obtained from Scopus using general pet food terms, including “pet food”, “dog food”, “cat food”, “canine”, “feline”, “companion animal nutrition”, and “companion animal food”, combined with precision and specialized nutrition keywords such as “precision nutrition”, “personalized”, “individualized”, “life-stage”, “senior”, “aging pet”, “breed-specific”, “hypoallergenic”, “obesity”, “renal”, “hepatic”, and “diabetic”.

Functional pet food research has increasingly embraced precision and specialized nutrition between 2016 and 2025, with a notable surge in publications after 2020 (Figure 11). This shift reflects the growing recognition that generalized dietary approaches are insufficient to meet the diverse physiological and pathological needs of a subset of companion animals. The rapid rise in studies focused on personalized and individualized nutrition mirrors advancements in human precision health, indicating a translation of omics-based and data-driven strategies into pet food development. Life-stage, senior, and aging pet nutrition have also gained prominence, driven by demographic changes such as increased pet longevity and owner demand for age-appropriate formulations that support musculoskeletal health, immune function, and cognitive maintenance. Breed-specific dietary research has shown steady growth, underscoring genetic variability in metabolism, body composition, and disease predisposition. Although hypoallergenic diets maintain a consistent niche within the literature, disease-targeted nutrition particularly for obesity has expanded markedly, reflecting the escalating prevalence of overweight and metabolically compromised pets [10,35]. Meanwhile, renal and hepatic dietary interventions, while less prolific, remain vital to clinical nutrition, requiring evidence-based formulation strategies under regulatory oversight [36]. Collectively, these trends underscore a movement toward individualized, health-optimized feeding paradigms within the functional pet food sector. The rapid growth of publications on precision and personalized pet nutrition reflects increasing conceptual alignment with human precision health frameworks. However, most commercially available pet foods continue to rely on predefined categories, such as life-stage, size, or condition-specific formulations, rather than truly individualized diets. While emerging studies explore genetic, metabolic, or microbiome-informed feeding strategies, their application in commercial products remains limited by data availability, cost, regulatory constraints, and validation requirements. Consequently, precision nutrition in the current pet food market is best characterized as an incremental extension of targeted nutrition rather than fully personalized dietary solutions.

## 4. Trends in Pet Food Innovation Patents

**Figure 12:**
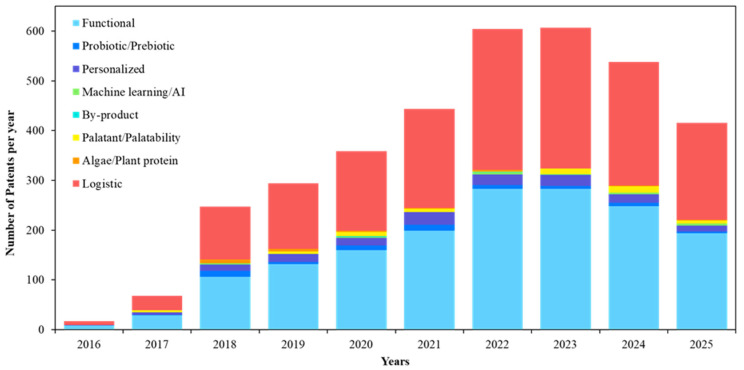
Annual number of pet food–related patent publications from the WIPO database from 2016 to 2025, classified by major technology themes. The data were obtained from WIPO PATENTSCOPE using general pet food terms, including “pet food”, “dog food”, “cat food”, “canine”, “feline”, and “companion animal food”, searched in English-language titles, abstracts, and claims together with theme-specific keywords related to functional health benefits, probiotics and microbiome, personalized or condition-specific nutrition, artificial intelligence and machine learning, by-product utilization, palatability, alternative proteins, and logistics or supply chain technologies. Patent publication dates were restricted to 2016–2025. Because keyword-based patent searches may not capture all relevant inventions and some patents may overlap across technology categories, the results should be interpreted as indicators of relative technological activity rather than comprehensive patent counts.

Trends in pet food innovation patents from the WIPO database during 2016–2025, using the specific search criteria shown in Figure 12, reveal a rising number of patent filings, particularly in functional food technologies and health benefits. These patents focus on formulas containing bioactive compounds or nutraceuticals aimed at supporting immunity, digestive health, skin and coat condition, joint health, cognitive function, antioxidant activity, and weight management. Patent activity in this category peaked in 2022–2023 before declining in recent years. Precision or specialized nutrition, including life-stage, breed-specific, and condition-targeted formulas such as hypoallergenic, obesity, renal, hepatic, or diabetic diets, showed a slow but steady increase in filings. The number of patents related to probiotics, prebiotics, and microbiomes remained low, primarily involving *Lactobacillus* or *Bifidobacterium* strains with known effects on digestive and immune function in pets. Technologies enhancing palatability, including flavor or aroma enhancers, Maillard reaction compounds, and hydrolysate-based formulations, accounted for only 55 patents during the period. Patents for alternative proteins in pet food, such as insect-based sources like *Hermetia illucens* (black soldier fly), were limited, while algae-derived proteins showed slightly higher activity. By-product utilization in pet food remains restricted, with a few notable filings, such as the use of egg by-products in polypeptide complexes as antibodies for canines and felines, and meat tenderizers derived from animal by-products applied in dry pet food extrusion processing. Logistics and supply chain–related patents represented a major technology category during the study period. The high level of activity suggests increasing industry interest in improving product stability, distribution efficiency, and commercialization of pet food products across global markets.

The limited number of patents in some areas, such as probiotics, personalized nutrition, machine learning/AI, and using by-products, shows there are issues with proving effectiveness, ensuring safety, scaling up production, and regulatory requirements. These constraints may have contributed to the limited commercial adoption of such technologies, despite sustained academic interest. Consequently, the slowdown or plateau observed in patent publication across certain categories may indicate industry adjustment to practical market constraints rather than a decline in the underlying technological relevance. Although patent data from the WIPO database demonstrate broad technological development in the pet food sector, the intensity of patent activity does not necessarily translate into commercial implementation. When looking at product launch data from Mintel’s Global New Products Database (GNPD), technologies that get to market quicker usually match better with what consumers want, how easy they are to make, everyday pet health needs, accepted safety proof, and product stability, instead of depending on very complicated or early-stage innovations.

**Figure 13:**
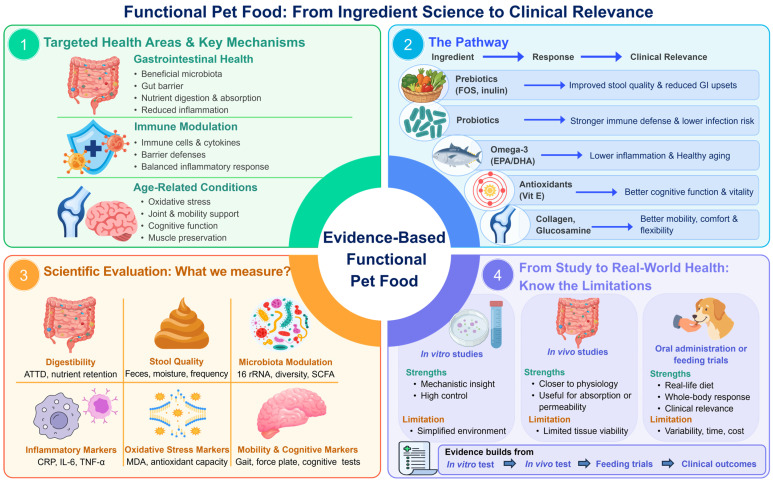
Conceptual framework linking ingredient functionality to physiological responses and clinical relevance in functional pet food research. The numbered and color-coded sections indicate the four main components of the framework, while the arrows represent the pathway from ingredients to physiological responses and clinical relevance. The framework provides a biological basis for interpreting how scientific findings may support subsequent technological development and commercial product innovation discussed in later sections.

Bibliometric analyses of Scopus and global patent landscapes indicate a clear transition in pet food innovation—from broad, general health claims toward mechanism-driven functional nutrition. Current research and patent activity are most intensely focused on formulations targeting gastrointestinal health, immune modulation, aging, obesity, skin and coat condition, and joint function. These domains are scientifically compelling because they enable direct linkage between ingredient functionality and measurable physiological endpoints, including digestibility, stool quality, microbiota modulation, inflammatory and oxidative stress biomarkers, as well as mobility and cognitive performance. In contrast, emerging approaches such as postbiotics, precision nutrition, AI-assisted formulation, and alternative protein systems remain comparatively underdeveloped, as their efficacy, stability, scalability, sensory acceptance, and regulatory robustness require further validation. Consequently, successful innovation in pet food systems depends not only on identifying bioactive ingredients but also on the capacity to translate mechanistic insights into products that are safe, stable, palatable, and compatible with industrial-scale manufacturing. This pattern suggests that innovation alone is a poor predictor of commercial success. Technologies that combine measurable biological efficacy with manufacturing compatibility, regulatory feasibility, and consumer acceptance are more likely to progress from scientific investigation to patent protection and market adoption. Figure 13 conceptualizes this framework by illustrating how health-targeted formulations are evaluated across multiple biological endpoints—while also acknowledging the inherent limitations and translational gaps associated with in vitro assays, in vivo models, and controlled feeding trials.

## 5. Mintel Product Launch Trend

**Figure 14:**
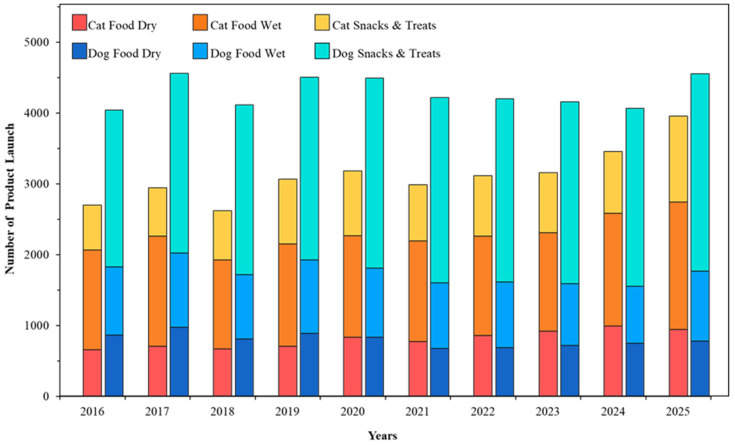
Number of new pet food products launched in Mintel’s Global New Products Database (GNPD) from January 2016 to December 2025, classified by pet type and product sub-category, including dry food, wet food, and snacks or treats for cats and dogs. The data were obtained from Mintel GNPD using the category match “Pet Food” and sub-category filters for “Cat Food Dry”, “Cat Food Wet”, “Cat Snacks and Treats”, “Dog Food Dry”, “Dog Food Wet”, and “Dog Snacks and Treats”. Product launch dates were limited to January 2016–December 2025. The product launch dataset was used as an indicator of commercial implementation and market adoption, enabling comparison with trends observed in scientific publications and patent activity.

Mintel GNPD data in Figure 14 for January 2016 to December 2025 show 74,427 new pet food launches with a clear upward trend. Total annual launches increased from 6741 in 2016 to the highest level of 8850 in 2025. Across 2016–2025, dog-related launches exceeded cat-related launches, driven mainly by Dog snacks and treats. Cat launches totaled 31,324 (42.1%), whereas dog launches totaled 43,103 (57.9%). This pattern suggests stronger innovation competition and faster portfolio turnover in the dog segment and aligns with consumer evidence from Morelli et al. (2021) [37], who reported a larger dog-owner sample than cat owners. Dog snacks and treats dominated the dataset with 25,590 launches (34.4%) and increased from 2213 in 2016 to 2901 in 2025. Cat food wet ranked second with 14,741 launches (19.8%) and remained a major contributor to market activity. Beyond launch volume, Morelli et al. (2021) [37] also showed that kibble feeding was the dominant practice and that many owners perceived preservatives as a potential health risk, underscoring the commercial relevance of product stability, packaging performance, and clean-label positioning. Consistent with this context, Samant et al. (2021) [9] described dry dog foods as a frequent purchase due to convenience and shelf stability, but with lower sensory acceptance than wet or semi-moist formats. This limitation increases reliance on palatants and aroma design, which helps explain why launch momentum is stronger in palatability-led segments such as dog snacks and treats than in the more mature dog dry food category. Cat snacks and treats and cat food dry showed slower growth overall and rebounded in 2025.

**Figure 15:**
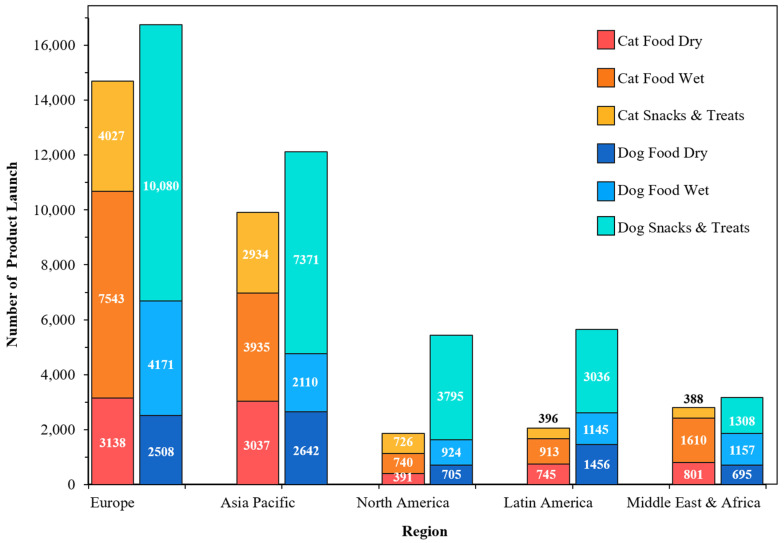
Number of new pet food products launched in Mintel’s Global New Products Database (GNPD) from January 2016 to December 2025, classified by geographic region, pet type, and product sub-category. Product sub-categories include dry food, wet food, and snacks or treats for cats and dogs. The data were obtained from Mintel GNPD using the category match “Pet Food”, regional market or import information, and sub-category filters for “Cat Food Dry”, “Cat Food Wet”, “Cat Snacks and Treats”, “Dog Food Dry”, “Dog Food Wet”, and “Dog Snacks and Treats”. Product launch dates were limited to January 2016–December 2025.

Mintel GNPD pet food product launches that were marketed or imported between January 2016 and December 2025 were summarized in Figure 15. The dataset includes 74,427 launches. Europe was the main hub with 31,467 launches (42.3%). Consistent with Europe’s leading share in our Mintel dataset, Prata [38] reported that commercial diets remain dominant, while interest in natural and organic alternatives is increasing, alongside greater scrutiny of ingredient lists and a preference for higher-meat and lower-carbohydrate formulations. Hoummady et al. [39] reported similar signals in France, including substantial uptake of non-conventional canine diets and frequent reliance on packaging guidance and online information, which supports demand for ingredient transparency and alternative-format products in Europe. Asia Pacific followed with 22,029 launches (29.6%). Consistent with this regional strength, Xiao et al. [40] showed that online pet food purchases in China are driven mainly by brand, ingredient transparency, and retailer credibility rather than import status, which can support faster product differentiation. Naseem Zahra [41] further described Pakistan as a fast-growing but import-reliant market, where higher import costs and limited domestic manufacturing favor shelf-stable formats and increase incentives for local production and functional positioning. Dog snacks and treats was the largest sub-category with 25,590 launches (34.4%). This segment was most pronounced in North America where it accounted for 52.1% of regional launches. The pattern indicates strong market pull for palatability-led formats and rapid functional claim turnover in treats. Cat food wet contributed 14,741 launches (19.8%). It concentrated in Europe and remained relatively high in the Middle East and Africa where it represented 27.0% of launches. This supports demand for high-moisture formulas and highlights the role of stability and packaging performance. Dog food dry was similar in overall scale to cat food dry but showed regional concentration. It represented 18.9% of launches in Latin America and reached its highest count in Asia Pacific. This aligns with cost efficiency and distribution advantages of shelf-stable dry products.

**Figure 16:**
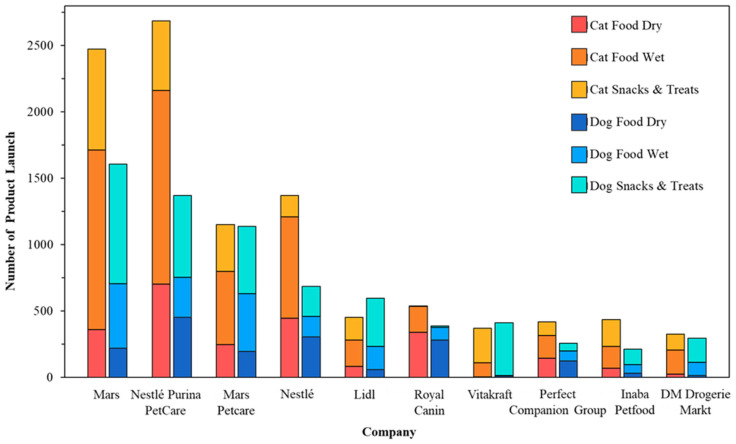
Number of new pet food products recorded in Mintel’s Global New Products Database (GNPD) from January 2016 to December 2025, classified by leading company or brand and product sub-category. Product sub-categories include cat dry food, cat wet food, cat snacks and treats, dog dry food, dog wet food, and dog snacks and treats. The data were obtained from Mintel GNPD using the category match “Pet Food”, sub-category filters for “Cat Food Dry”, “Cat Food Wet”, “Cat Snacks and Treats”, “Dog Food Dry”, “Dog Food Wet”, and “Dog Snacks and Treats”, and product records marketed or imported by region. Product launch dates were limited to January 2016–December 2025. Company-level launch activity was analyzed as an indicator of technology translation and commercialization intensity within the pet food sector.

Figure 16 summarizes the distribution of pet food product launches recorded in Mintel’s Global New Products Database (GNPD) from January 2016 to December 2025, comprising a total of 74,427 launches. Analysis of this dataset indicates that Mars led the market with 4079 product launches, followed closely by Nestlé Purina Pet Care with 4055 launches. Mars Petcare ranked next with 2292 launches, while Nestlé accounted for 2057 launches during the same period. Together, these four producers accounted for about 16.8% of all launches, indicating innovation competition driven by line extension and reformulation rather than strong market concentration. Dog snacks and treats were the primary battleground, totaling 25,590 launches, with Mars leading this segment at 900 launches. Treat-focused players such as Vitakraft (398) and private-label retailers such as Lidl (386) and DM Drogerie Markt (182) also contributed materially to launch volume. Cat food wet accounted for 14,741 launches and showed high brand density, led by Nestlé Purina (1458) and Mars (1352), consistent with strong demand for palatability and product stability that relies on formulation and packaging performance. As contextual support, Buckley and Chavez [42] describe the pet food sector as being led by large multinationals, with Mars Petcare positioned as the dominant firm, followed by Nestlé Purina PetCare. They also highlight Mars’s extensive portfolio across major pet food brands (e.g., Pedigree, Whiskas, Royal Canin) and veterinary service chains (e.g., Banfield, VCA, Pet Partners), indicating influence that extends beyond manufacturing. For dry diets, Royal Canin was prominent in cat food dry (338) and dog food dry (282), reflecting condition-specific nutrition and health benefit positioning. Overall, treats and wet foods appear to be faster channels for innovation and claim cycling, whereas dry foods compete through quality consistency, specialized formulations, and manufacturing efficiency.

Health-oriented functional ingredients, digestive health solutions, and palatability-focused technologies show strong representation across all three datasets, suggesting relatively successful translation from research to market. In contrast, postbiotics, artificial intelligence–based nutrition systems, and advanced precision nutrition concepts exhibit growing publication activity but remain less visible in patent and commercial launch data, indicating continuing translational barriers.

## 6. Technology Drivers and Translational Innovation Clusters in Pet Food

**Figure 17:**
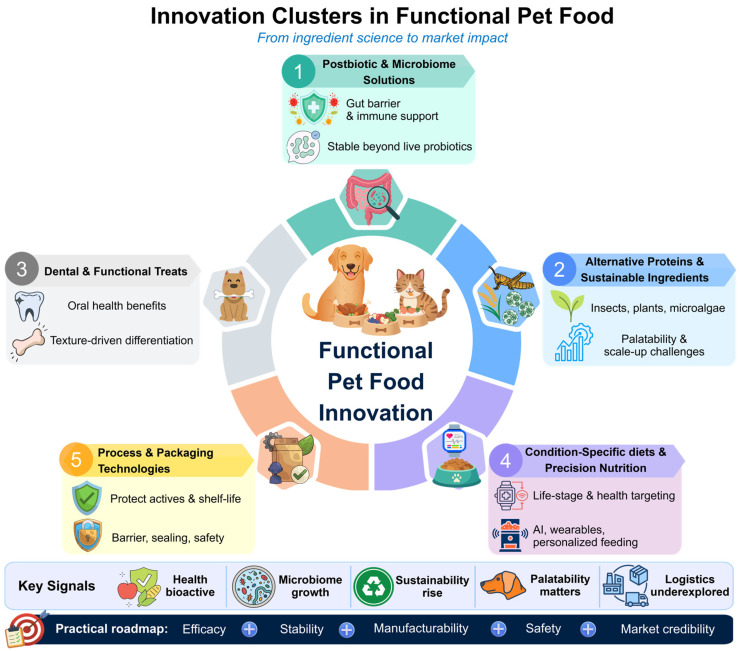
Key innovation clusters in pet food.

Mintel GNPD launch trends indicate that growth in the pet food sector is not driven by health positioning alone, but by technologies capable of translating health concepts into products that are manufacturable, stable, and commercially credible. Consistent with this trend, Vuthisopon et al. [43] show that functional pet food innovation is increasingly directed toward specific physiological targets, including digestive health, joint mobility, cognitive support, and behavioral well-being. Figure 17 organizes these developments into five innovation clusters that capture where research and commercialization are converging.

### 6.1. Postbiotic and Microbiome Solutions

Postbiotic and microbiome solutions may support the idea of a bifidogenic effect and barrier function, anti-inflammatory signaling, and immune modulation in animals [44]. Postbiotics offer greater stability than probiotics and may retain functional value without the limitations associated with microbial viability and intestinal colonization, thereby addressing an important industrial feasibility gap in pet food production [45]. Their preparation and incorporation are also more compatible with high-temperature feed and pet food processing than live microbial systems, which increases their practical relevance for commercial applications [45]. Bonel-Ayuso et al. [46] synthesized canine postbiotic studies and reported that pooled effects were not statistically different from controls, largely due to small samples and high heterogeneity in postbiotic types and study designs. This evidence gap supports innovation emphasis on processing stability, delivery technologies, and condition-targeted microbiome positioning. Wernimont et al. [47] frame the canine and feline GI microbiome as a metabolically active organ shaped by diet, with potential links to both GI and extra-intestinal conditions, including allergy, weight management, diabetes, and kidney disease. They highlight a shift from describing microbiome composition to quantifying microbiome function using metabolites and other functional readouts, which supports current innovation emphasis on stable, condition-targeted microbiome and postbiotic solutions. Zhu et al. [48] identified several challenges in the use of postbiotics in animal and pet food, including the need for clearer characterization of active components, optimized dosage and specifications, the absence of unified efficacy evaluation criteria, and limited clinical evidence. G. R and V [49] further identified constraints in industrial-scale production, particularly in maintaining stability, quality, yield, and consistency. These issues suggest that successful translation of postbiotics into marketable products depends on both biological promise and reliable industrial manufacture.

Gut health technologies provide a clear example of the link between scientific research and market development in pet food. However, commercial success depends not only on biological function but also on processing stability. Acuff and Aldrich [50] explains that the thermal and mechanical forces during extrusion and drying are major challenges for maintaining probiotic viability in pet food production. Rodrigues et al. [51] suggest that pet food manufacturing processes designed to improve food safety and prolong shelf-life may also reduce the survival of direct-fed microbials (probiotics) during storage and simulated gastrointestinal passage, highlighting the critical nature of technological robustness.

This cluster also overlaps with immune-modulating pet food technologies, which represent another clear example of how scientific rationale supports market development. These products are more often positioned around resilience, defense, or wellness than disease treatment, which supports the commercial use of beta-glucans, yeast derivatives, antioxidants, omega-3 fatty acids, and other bioactive ingredients [43,52]. Scientifically, these ingredients may support immune function through mechanisms such as oxidative stress reduction, mucosal immune support, modulation of inflammatory mediators, and gut–immune interactions [53,54]. This provides a more specific basis for immune support than vague claims to “boost immunity. Boothe [55] further emphasized that scientific evidence on manufacturing quality assurance, safety, and efficacy to build consumer confidence in novel ingredients, which translates biological function into credible, marketable products.

### 6.2. Alternative Proteins and Sustainable Ingredients

Alternative proteins and sustainable ingredients respond to growing sustainability expectations while offering the potential to reduce environmental burdens relative to conventional animal-derived proteins. Current work emphasizes insect–plant blends and tackles two barriers that determine adoption. These barriers are palatability and digestibility. Klinmalai et al. [56] integrate publications, WIPO patent signals, and Mintel GNPD product trends on plants, aquatic, insect, and cell-based proteins. They highlight that adoption is constrained mainly by palatability and digestibility, while product strategies often rely on blends and low-level functional additions, with notable commercialization signals for microalgae and established use of insect proteins in pet foods. This synthesis aligns with our ‘Alternative Proteins’ cluster and supports an innovation focus on sensory engineering and processing to enable wider uptake. Consumer interest also supports their market relevance, as pet owners increasingly prefer ingredients associated with ethical sourcing and sustainable production [57]. However, successful commercialization depends on more than sustainability appeal alone. For example, scaling up cultivated meat remains constrained by challenges related to bioreactor capacity, environmental control, and contamination prevention before production can meet industrial and commercial demands [25]. Gordon et al. [58] further emphasized that biomaterials for cellular agriculture and plant-based foods must function at scale, at low cost, and within food safety requirements, highlighting a persistent technological and economic barrier to commercialization.

### 6.3. Dental and Functional Treats

Dental and functional treats represent a high-velocity format for innovation. Product differentiation often relies on design features and utility claims. Texture engineering is central because plaque removal depends on mechanical interaction during chewing. Consistent with this market dynamic, Calancea et al. [59] describe dog treats as a rapidly expanding category driven by owner–dog bonding and preferences for meat-forward “natural” positioning, while also highlighting label clarity gaps and the need to manage treat calories within total daily intake. Clinical support for utility claims is also available Carroll et al. [60] reported that daily dental chews reduced plaque and calculus and lowered breath volatile sulfur compounds versus no-chew controls, indicating that chew architecture can deliver measurable oral-health benefits.

### 6.4. Condition-Specific Diets and Precision Nutrition

Condition-specific diets extend precision nutrition into mainstream portfolios. Priority areas include weight management, renal support, and skin and coat formulas. Clinical backing is increasingly required to justify health positioning and to support veterinary and evidence-based recommendations. This approach aligns with clinical nutrition guidance that recommends individualized feeding plans based on life stage and environment, with body condition as a primary target and treats capped at a small share of daily calories to protect weight control. Fascetti and Delaney [61] also highlight treat safety considerations, reinforcing the need for evidence-led product framing. For skin/coat claims, Connolly and Wu [62] provide a mechanistic rationale by linking tissue structure to targeted amino acid supply for keratin, collagen, and elastin, with quantitative needs shaped by breed, age, and coat characteristics. Leverett et al. [63] provide intervention data in healthy client-owned dogs showing that switching between fresh and dry diets alters the skin microbiome; alpha diversity was higher during fresh feeding and community composition shifted, supporting skin/coat innovation that can be anchored to measurable microbiome endpoints.

Healthy aging and condition-specific nutrition involve a convergence between technology and market evolution in pet food. Current life-stage, breed-specific, and therapeutic nutrition frameworks support more individualized dietary strategies for conditions such as renal insufficiency, obesity, cognitive decline, and dermatological disorders [64]. These approaches include phosphorus restriction for chronic kidney disease, medium-chain triglyceride enrichment for cognitive support, and selected fatty acids or antioxidants to support joint, skin, and healthy aging functions [43,64]. Churchill and Eirmann [65] further highlighted a discrepancy between pet owners perceived nutritional needs for senior dogs, including fewer calories and lower fat, sodium, protein, and carbohydrate contents, and the actual composition of many commercially marketed senior diets, underscoring the need for closer alignment between scientific formulation and market positioning.

Precision nutrition and digital pet health tools may play an increasingly important role in optimizing ingredient formulation, strengthening quality control, and enabling more personalized nutrition in the pet food industry. Kumar and Sharma [66] suggest that machine learning can support ingredient selection, real-time quality monitoring, predictive health analytics, and consumer preference modeling. AI-based systems may also help address obesity and related disorders by automating feed calculations from health-related data such as body weight, temperature, behavior, and heart rate [67]. However, wider adoption is still limited by device cost, data privacy concerns, reliability, connectivity, and compliance with regulations such as the Personal Data Protection Act (PDPA) [68].

### 6.5. Process and Packaging Technologies

Process and packaging technologies act as enabling platforms across all clusters. Coatings are used to protect actives and to control release. Oxygen and moisture barriers are used to preserve sensory quality and nutrient stability. Recent packaging analytics highlight safety as a parallel constraint. Chinthakindi et al. [69] detected low but measurable perfluoroalkyl acids in plastic pet food packaging and matched foods, and higher post oxidation perfluorocarboxylic acids (PFCAs) levels suggested the presence of precursor compounds. This supports poly- and perfluoroalkyl substances (PFAS) aware barrier selection and routine monitoring. Wagoner et al. [70] showed that sodium alginate combined with encapsulated calcium lactate helped maintain surface color and moderate lipid oxidation in vacuum packaged co product diets during a 21 day refrigerated display. This demonstrates how ingredient technology and packaging jointly manage shelf-life. Pain et al. [71] reported lead residues in dog foods containing wild shot pheasant, with many raw products exceeding EU feed limits. The pattern was consistent with shot fragments that are further dispersed by mincing. This strengthens the case for contaminant surveillance and non-lead sourcing in meat from wild-shot game diets. White et al. [72] argue that newer processing and packaging formats change the hazard profile. The shift from cans to retortable pouches and trays adds sealing and thermal validation challenges. This supports tighter process control and pack integrity validation as formats diversify. These clusters define a practical roadmap for linking ingredient science to manufacturability and to market impact. The three datasets show how pet food innovation develops from research to commercial products. Scientific publications mainly focus on health functions, biological mechanisms, and new ingredients. Patents reflect efforts to develop these findings into technologies that can be produced, protected, and applied in pet food products. Product launch data show which technologies have reached the market successfully. Digestive health, immune support, palatability technologies, and some alternative proteins show strong activity across publications, patents, and product launches. In contrast, postbiotics, AI-based nutrition tools, and advanced precision nutrition have received increasing research attention but remain less visible in patents and commercial products. These differences suggest that some technologies are more difficult to develop into market-ready products than others.

The publication and patent landscape in functional pet food research shows uneven development across themes, with the strongest concentration in health-oriented functional foods and bioactive ingredients. A second major body of work centers on microbiome modulation, where probiotics and prebiotics dominate, while postbiotics and broader microbiome-driven strategies appear less frequent [73]. AI, machine learning, and predictive modeling are present at moderate levels, implying a growing role as enabling tools for formulation optimization rather than core innovation drivers. Sustainability-related work is increasingly visible but is framed more around upcycling and waste valorization than fully articulated circular-economy systems [74]. Sensory technologies such as palatants and coatings occupy a mid-range position, underscoring the importance of palatability in ensuring adoption of health-promoting formulations, while more specialized chemistry-based flavor routes appear less common [75]. Finally, alternative proteins show rising but uneven attention: insect protein stands out, while microbial and fermentation-derived proteins remain underexplored [56], and logistics-focused topics are least represented, suggesting innovation efforts are still concentrated upstream on ingredients and formulation rather than downstream supply-chain optimization.

Across the innovation clusters reviewed, translational barriers differ substantially according to technology maturity. For example, postbiotic applications remain constrained by limited standardization and clinical validation [48,49], while probiotic systems face challenges associated with microbial survival during processing and storage [50,51]. Alternative proteins and cultivated meat are primarily limited by production scalability, manufacturing costs, and regulatory approval pathways [25,58]. In contrast, AI-based nutrition platforms are constrained by data availability, implementation costs, privacy concerns, and regulatory compliance requirements [66,67,68]. These observations suggest that successful commercialization depends not only on biological efficacy but also on overcoming technology-specific barriers that influence industrial feasibility and market adoption.

## 7. Conclusions

The integrated analysis of scientific publications, patent activity, and global product launches reveals that innovation in the pet food sector is advancing unevenly across domains. Health-oriented functional formulations remain the most mature area, with sustained emphasis on digestive health, immune modulation, and age-related conditions. In contrast, postbiotics, microbiome-driven strategies, precision nutrition, and data-enabled approaches such as artificial intelligence are still underdeveloped in terms of validation, scalability, and commercial deployment. Sustainability-related innovation is gaining momentum, particularly through alternative proteins and by-product utilization. However, most efforts remain focused at the ingredient level, while fully integrated circular economic systems continue to face technological, regulatory, and logistical barriers. Similarly, sensory and palatability technologies remain essential enablers of adoption, underscoring that functional efficacy alone is insufficient without consumer and animal acceptance. A persistent gap remains between research activity, intellectual property development, and market implementation. The findings further indicate that scientific activity, patent generation, and market adoption do not progress at equivalent rates. Instead, successful commercialization appears to depend on the ability to overcome translational barriers related to efficacy validation, process stability, regulatory acceptance, and economic scalability.

Addressing this gap will require multidisciplinary strategies that combine mechanism-based nutritional science, robust safety and efficacy validation, regulatory alignment, and manufacturing feasibility. However, the advancement of such research remains strongly influenced by the availability of sustained funding, with limited financial support in academia often representing a critical bottleneck. Strengthened collaboration among academia, industry, and public funding agencies is therefore essential to enable translational research and long-term innovation. These innovation clusters demonstrate that the potential to combine biological mechanisms with processing stability, formulation accuracy, and industrial feasibility has become more important for pet food innovation than functional concepts alone. The most important innovations in this context are probably innovations that convert scientific data into reliable, scalable, and feasible for production technology. Future progress in pet food innovation will depend less on introducing new functional concepts and more on integrating existing advances into scalable, compliant, and market-ready solutions. The science, patent and market framework presented in this review provides a structured basis for identifying these translational priorities and guiding next-generation pet nutrition development.

## Data Availability

The data presented in this study is available on request from the corresponding author.
